# Hyperglycaemia is inversely correlated with live M. bovis BCG‐specific CD4^+^ T cell responses in Tanzanian adults with latent or active tuberculosis

**DOI:** 10.1002/iid3.222

**Published:** 2018-04-11

**Authors:** Noémie Boillat‐Blanco, Anneth‐Mwasi N. Tumbo, Matthieu Perreau, Patrizia Amelio, Kaushik L. Ramaiya, Maliwaza Mganga, Christian Schindler, Sebastien Gagneux, Klaus Reither, Nicole Probst‐Hensch, Giuseppe Pantaleo, Claudia Daubenberger, Damien Portevin

**Affiliations:** ^1^ Ifakara Health Institute Dar es Salaam United Republic of Tanzania; ^2^ Swiss Tropical and Public Health Institute Basel Switzerland; ^3^ Department of Sciences University of Basel Basel Switzerland; ^4^ Infectious Diseases Service Lausanne University Hospital Lausanne Switzerland; ^5^ Division of Immunology and Allergy Lausanne University Hospital Lausanne Switzerland; ^6^ Shree Hindu Mandal Hospital and Muhimbili University of Health Sciences Dar es Salaam United Republic of Tanzania; ^7^ Kinondoni Municipal Council National Tuberculosis Program Dar es Salaam United Republic of Tanzania

**Keywords:** Adaptive immunity, diabetes, hyperglycaemia, tuberculosis

## Abstract

**Introduction:**

The rising prevalence of Diabetes mellitus (DM) in high TB‐endemic countries may adversely affect sustainability of TB control since DM constitutes a risk factor for development of active tuberculosis (TB). The impact of DM on TB specific adaptive immune responses remains poorly addressed, particularly in people living in Sub‐Saharan countries. We performed a functional characterization of TB specific cellular immune response in Tanzanian subjects with active or latent *Mycobacterium tuberculosis (Mtb)* infection stratified by their diabetic status.

**Methods:**

HIV negative active TB patients (≥18 years) with Xpert MTB/RIF positive pulmonary TB were included before starting TB treatment in Dar es Salaam, Tanzania between April and December 2013. HIV negative healthy controls latently infected with TB but without past TB history were also included. Active and latent TB patients were stratified in two groups according to their diabetic status. Peripheral Blood Mononuclear cells were stimulated with either live *M. bovis* BCG or *Mtb*‐specific peptide pools and analyzed by intracellular cytokine staining and polychromatic flow cytometry.

**Results:**

Our results show a lower frequency of IFN‐γ CD4^+^ T cells in patients with active TB and DM compared to patients with active TB only after live *M. bovis* BCG (*p* = 0.04) but not after *Mtb* peptide pools re‐stimulation. Irrespective of TB status, level of glycaemia is selectively inversely correlated with IFN‐γ and TNF‐α CD4^+^ T cell production (*p* = 0.02 and *p* = 0.03) after live *M. bovis* BCG stimulation.

**Conclusions:**

These results support the hypothesis that hyperglycaemia negatively impacts antigen processing and/or presentation of whole mycobacteria delaying secretion of key cytokines involved in TB immunity.

## Introduction

Diabetes mellitus (DM) increases the risk of active tuberculosis (TB) by about threefold 1. The recent rise of type 2 DM prevalence in countries with pre‐existing high TB burden is expected to negatively impact current TB control efforts 2. The interaction between active TB and DM suggests that host defence mechanisms for control of *Mycobacterium tuberculosis* (*Mtb*) infection might be impaired. Hyperglycaemia affects biological functions of neutrophil granulocytes, macrophages and natural killer cells, important constituents of the innate immune system 3. T cell‐mediated immune responses are clearly critical in the outcome of *Mtb* infection as demonstrated by the marked susceptibility and reactivation of TB in HIV‐co‐infected persons 4. Defects in pro‐inflammatory CD4^+^ T cell polarization and cytokine production, particularly IFN‐γ, are well‐defined risk factors for mycobacteriosis 5. The immunological consequences of DM co‐morbidity on TB specific adaptive immunity has been evaluated in different human studies with conflicting results 6. Increased production of Th1 cytokines following PBMC stimulation with *Mtb* derived purified protein derivative in DM and TB co‐morbid patients compared to non‐DM TB patients has been observed in India, Mexico, and Texas, but not in Indonesia 7, 8, 9.

To our knowledge, the immunological hallmarks underlying the negative impact of DM on TB disease progression in sub‐Saharan Africa have not been analyzed in depth. We performed functional characterization of CD4^+^ T cells in active and latent TB rigorously stratified by their diabetic disease status. In contrast to previously published studies, we stringently classified our DM cases based on three recommended screening tests that were repeated 5 months after TB treatment initiation to exclude patients with transient stress‐induced hyperglycaemia 10. To compare antigen‐specific memory T cell frequencies across our study participants, PBMC were stimulated with live *M. bovis* BCG (requiring antigen processing and presentation) or *Mtb*‐specific peptide pools (not depending on antigen processing and presentation) followed by intracellular cytokine staining and polychromatic flow cytometry. This comparison has to our knowledge not been assessed systematically in TB DM comorbidity studies.

## Results and Discussion

### Study cohort

**Figure 1:**
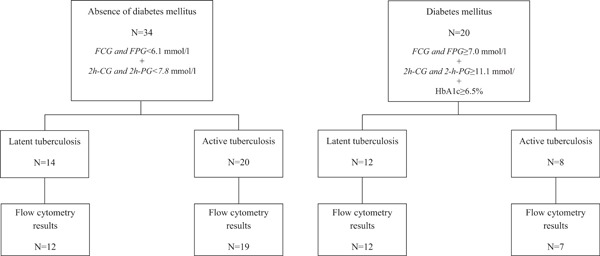
Flow chart of study participants and availability of flow cytometry results. Definitions: FCG: fasting capillary glucose/FPG: fasting plasma glucose/2h‐CG: two‐hours capillary glucose/2h‐PG: 2‐hplasma glucose/HbA1c: glycated haemoglobin.

We enrolled 26 volunteers with latent TB and 28 active TB cases. Study participants with DM were significantly older compared to normoglycaemic volunteers (Table 1). Overall, 94% of study participants were previously vaccinated with BCG. Figure 1 depicts the distribution of participants with latent TB and active TB according to their diabetic status. Our study population was rigorously characterized for their DM status based on three currently recommended screening tests that were repeated 5 months after TB treatment initiation. We therefore excluded all patients with transient stress‐induced hyperglycaemia that returned to normal after TB treatment 10, 11. Despite systematic screening of TB patients for DM, the recruitment of patients with active TB and DM was greatly impeded by exclusion of individuals with HIV co‐infection and of TB patients with transient hyperglycaemia that does not represent true DM. Among patients with active TB and DM co‐morbidity, 5 out of 8 were treated for DM prior to TB diagnosis (4 were treated with a combination of metformin and glibenclamide and 1 with insulin). Metformin, which is frequently used for DM treatment, facilitates phagosome‐lysosome fusion, and enhances *Mtb*‐specific immune response 12. Glibenclamide that was also widely used in our study population has a wide range of anti‐inflammatory effect on the host inflammasome 13. All volunteers with latent TB and DM comorbidity were reported to be under DM treatment at the time of blood collection (1 was treated by metformin, 10 by a combination of metformin and glibenclamide and 1 received insulin). However, blood glycaemia levels were clearly not controlled in these DM cases suggesting either poor treatment adherence and/or interference of tuberculosis itself with glycaemic control and treatment (Table 1).

**Table 1:** Study population

	Latent Mtb *N *= 14	Latent Mtb DM *N *= 12		Active TB *N* = 20	Active TB DM *N* = 8	
	*N* (%)/median (IQR)		*p* value	*N* (%)/median (IQR)		*p* value
Age	35 (10)	45 (27)	<0.001	28 (11)	57 (7)	<0.001
Male sex	10 (71)	5 (42)	0.13	17 (85)	7 (88)	0.86
Low socio‐economic status	3 (23)	2 (17)	0.69	7 (37)	3 (43)	0.78
Body mass index (kg/m^2^)	23.4 (5.5)	26.3 (8.8)	0.96	18.4 (4.3)	18.7 (3.6)	0.57
Under treatment for diabetes	–	12 (100)	–	–	5 (63)	–
TB symptom duration >1month	–	–	–	15 (79)	6 (86)	0.70
Fasting capillary glucose (mmol/L)	4.8 (0.7)	10.1 (4.6)	<0.001	5.0 (0.5)	18.3 (12)	<0.001
Fasting plasma glucose (mmol/L)	5.1 (1.1)	16.4 (4.6)	<0.001	5.1 (0.6)	20.7 (6.2)	<0.001
Glycated Hemoglobin (%)	5.4 (0.6)	11.2 (3.4)	<0.001	5.8 (0.7)	13.6 (4.5)	<0.001

Latent *Mtb*: *Mycobacterium tuberculosis* latent infection defined as positive *Mtb*‐specific Interferon‐Gamma enzyme‐linked immunospot assay or positive *Mtb*‐specific flow cytometry response/TB: tuberculosis/DM: diabetes mellitus. *p* value calculated with two‐sided Wilcoxon–Mann–Whitney or chi‐square tests when appropriate.

### Frequencies of IFN‐γ and TNF‐a producing Mtb‐specific CD4^+^ T in relation to blood glucose levels

**Figure 2:**
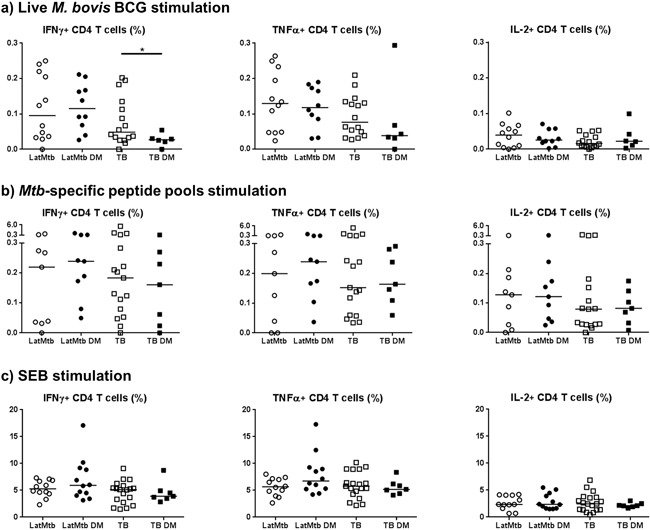
Frequencies of CD4^+^ T cells producing cytokines between the different study groups after: (a) live *M. bovis* BCG stimulation; (b) *Mtb*‐specific peptide pool stimulation; (c) SEB stimulation.

Following live *M. bovis* BCG stimulation of PBMC, we used intracellular cytokine staining and polychromatic flow cytometry to describe the quality and quantity of *Mtb*‐specific immune responses in our cohort (Supplementary Fig. S1). We observed a reduced frequency of IFN‐γ producing CD4^+^ T cells (*p* = 0.04) in TB cases with DM compared to normoglycaemic TB cases (Fig. 2a). In contrast, after *Mtb*‐specific peptide pools stimulation, no difference in cytokine producing CD4^+^ T cells frequencies was observed between study groups (Fig. 2b). Polyclonal SEB stimulation was performed as positive control on all samples analyzed and no difference in frequencies of IFN‐γ, TNF‐α, or IL‐2 producing CD4^+^ T cells was measured (Fig. 2c).

**Figure 3:**
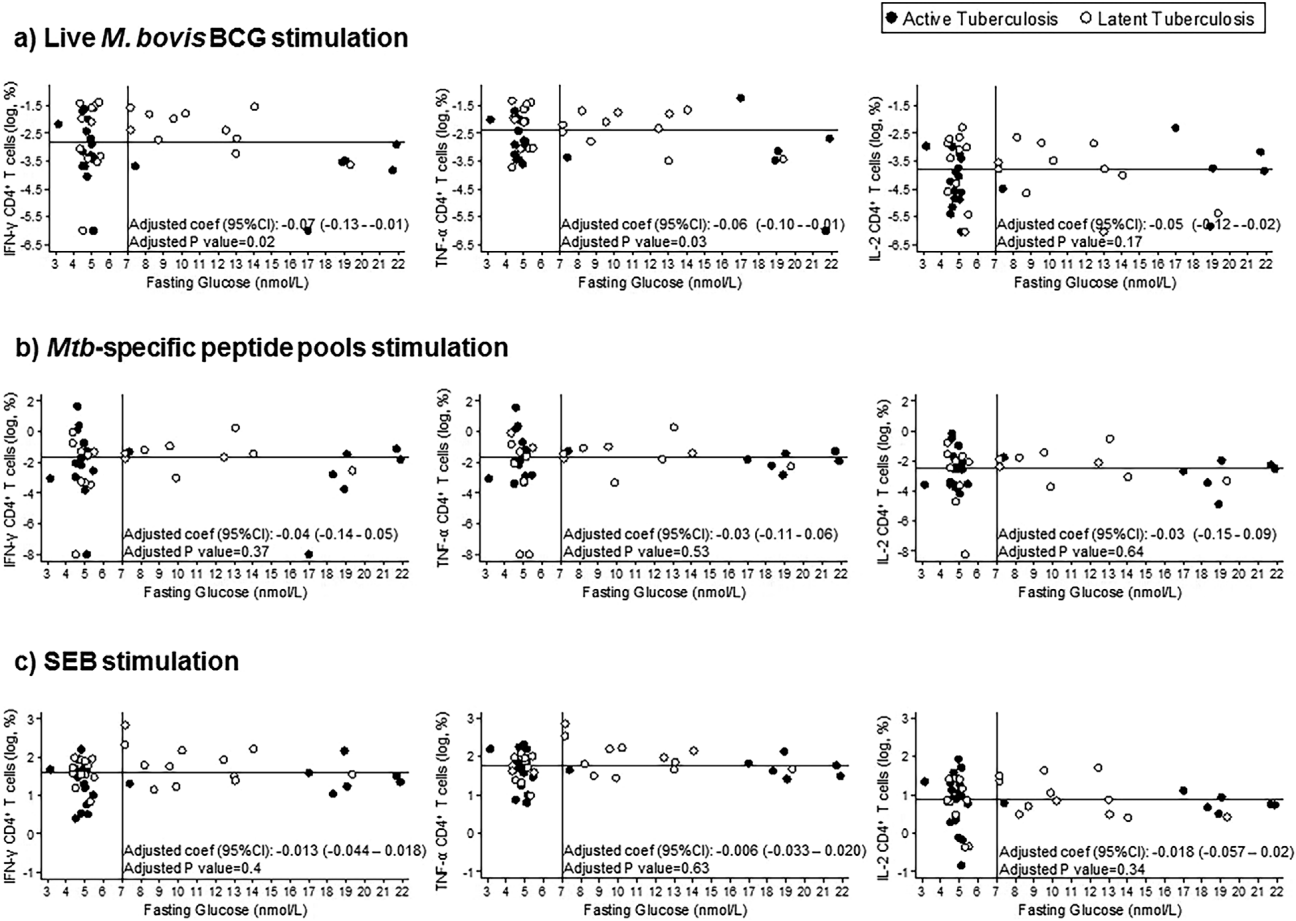
Dot‐plot graphs of the frequencies of CD4^+^ T cells producing cytokines against fasting capillary glucose levels after: (a) live *M. bovis* BCG stimulation; (b) *Mycobacterium tuberculosis*‐specific peptide pool stimulation; (c) Staphylococcal enterotoxin B stimulation. Statistical analysis: Linear regression adjusted for age and sex. The horizontal line represents the median value of the logarithmic frequency of CD4^+^ T cells producing cytokines and the vertical line represents the fasting glucose level cut‐off for DM disease classification. Patients with active tuberculosis: *n* = 28. Patients with latent tuberculosis: *n* = 26.

Given the limiting sample size that may affect the power of our study when patients are stratified, we next investigated whether the level of intracellular cytokine production of *Mtb*‐specific CD4^+^ T cells would correlate with DM disease severity irrespectively of TB disease status. An increase in glycaemia was found to be negatively correlated with IFN‐γ and TNF‐α CD4^+^ T cells frequency after live *M. bovis* BCG stimulation even after adjustment for age and sex (adjusted *p* = 0.02 and 0.03, respectively). IL‐2 production was not affected by the blood glucose levels (adjusted *p* = 0.17). In contrast, no difference after *Mtb*‐specific peptide pool or SEB control stimulation was seen (Fig. 3).

Our study has several strengths when compared to previously published data. First, our study population is very well defined and has been followed longitudinally for 5 months 10. Second, PBMC collection was performed prior to TB treatment initiation since cellular immune responses change rapidly under TB treatment 4. Third, we have studied PBMC instead of whole blood to circumvent potential cofounders in relation to hyperglycaemia during the whole blood assay 14. Fourth, we compared two different PBMC stimulation approaches using whole live *M. bovis* BCG side by side with ESAT6 and CFP10 peptide pools stimulation. Despite more than 99.95% identity 15, *M. bovis*, and its derivative, *M. bovis* BCG, underwent significant genomic deletion events including virulence factors when compared to most clinical *M. tuberculosis* isolates. Comparative genome analysis showed that out of 483 experimentally verified human T‐cell epitopes targeting *M. tuberculosis*, 329 were present in the genome of the BCG Danish strain used in this study 16. Therefore, *M. bovis* BCG expresses a substantial breadth of T‐cell antigens overlapping with *M tuberculosis* cellular immune targets. Antigen processing of whole mycobacteria‐derived antigens for MHC class II presentation on the surface of antigen presenting cells requires phagocytosis, processing, and intracellular vesicular trafficking to allow the formation of peptide‐MHC‐II complexes 17. Soluble, exogenous synthetic peptides can be directly loaded onto MHC class II molecules present on the surface of antigen‐presenting cells and presented to CD4^+^ T cells 18, 19. Head to head comparison of synthetic peptides versus whole‐mycobacteria to recall immune memory in vitro distinguishes between ex vivo assessment of CD4^+^ T cell frequencies directed against a specific antigen from the capacity of antigen‐presenting cells to uptake bacteria, process and present bacteria‐derived antigens to CD4^+^ T cells.

To our knowledge, one study examined the influence of DM on CD4^+^ T cells responses from active TB cases after stimulation with various *Mtb* antigens including purified protein derivative or ESAT6 and CFP10. Contrasting with our observations, higher frequencies of CD4^+^ T cells expressing single or combinations of IFN‐γ, IL‐2, and TNF‐α were found in active TB with DM compared to non‐diabetic TB cases, suggesting a sustained, strong adaptive immune response in the presence of DM co‐morbidity 7. This study reported higher frequencies of CD4^+^ T cell producing cytokines without stimulation (baseline) than under antigen specific stimulation 7. Our gating and analytical strategy used here is fundamentally different since T cell signals induced after antigen stimulation were actually corrected by background signals observed in the negative controls. We cannot exclude that the use of cryopreserved PBMCs may have preferentially affected antigen presentation of monocytes from hyperglycaemic patients. Inclusion of a viability dye in the flow cytometry analysis and gating strategy indicated an overall excellent cell viability across all samples (IQR: 98.6–95.8) which was not significantly affected by DM status (IQR: 98.4–95.7). Another study assessed secretion of IFN‐γ, IL‐2, TNF‐β, IL‐4, IL‐5, and IL‐10 into cell culture supernatants after stimulation with purified mycobacterial antigens or whole‐mycobacteria. No significant differences among patients with active TB and DM were described 20. Collectively, these results and ours suggested that *Mtb*‐specific adaptive immunity is not suppressed by DM but either unaffected or enhanced. However, the fact that the negative correlation between IFN‐γ and TNF‐α CD4^+^ T cells frequency and DM severity was observed under live *M. bovis* BCG stimulation is consistent with the defective functions of antigen‐presenting cells among diabetics and patients with TB and DM 21, 22, 23 and may promote delayed onset of the adaptive immune system that could contribute to TB DM co‐morbidity.

Different factors related to the host and pathogen genetics and to the selection of the study participants make comparison of the published results difficult. The study of Kumar et al. was conducted in India and DM diagnosis was based on glycated haemoglobin or random blood glucose measured at TB diagnosis while Al‐Attiyah et al. included TB patients in Kuwait and defined DM using two blood glucose measurements 7, 20. In our study, patient stratification according to DM was the most stringent possible using repeated fasting glucose, an oral glucose tolerance test and glycated haemoglobin assays. DM status was further confirmed 5 months after TB treatment initiation to avoid inclusion of patients presenting with transient stress‐induced hyperglycaemia 10. In addition, all pre‐DM patients were excluded during enrollment. Most of our diabetic participants were previously diagnosed and treated for DM and did not take their DM treatment the day before study inclusion. We were unable to trace this DM treatment information in other published studies. We would like to propose that our stringent DM classification could be followed in future to clarify the interaction of DM and immune responses to infectious agents across different study populations. Futures studies should also describe the impact of the different DM treatment medications on the Mtb specific immune responses as they might contribute as host directed therapy to tuberculosis containment 12, 24.

### Concluding remarks

A significant negative correlation existed between increasing levels of hyperglycaemia and the ability to measure IFN‐γ and TNF‐α producing CD4^+^ T cell responses following live *M. bovis* BCG stimulation. This observation is in line with experimental studies showing defective functions of antigen‐presenting cells among diabetics and with the reduced interaction of *Mtb* with monocytes among patients with TB and DM 21, 22, 23. Our results clearly warrant further investigation of the impact of blood glucose concentrations on antigen processing and presentation of whole live mycobacteria, notably in the context of the great potential of testing Metformin as host‐directed adjunct therapy during TB drug treatment that may specifically compensate for this defect 12.

## Materials and Methods

### Study design and setting

This prospective case‐control study was nested within an epidemiological study on the association between TB and DM 10. Study participants were recruited in Kinondoni District in Dar es Salaam (Mwananyamala Regional Hospital, Sinza Hospital, Magomeni Health Centre, and Tandale Dispensary). We planned for recruitment of 20 patients per study group to arrive at a first overview of the impact of DM on *Mtb*‐specific adaptive immune responses. Patients with active TB (with or without DM) were recruited between April and December 2013 and latent TB cases (with and without DM) between May and October 2013.

### Study participants

Active TB patients (≥18 years) with microscopy smear positive pulmonary TB were screened for inclusion. Patients were eligible if they were not on TB treatment, had a positive sputum Xpert MTB/RIF (Cepheid, Sunnyvale) assay and were HIV negative (Alere Determine™ HIV‐1/2 and Trinity Biotech Uni‐gold™ Recombigen® HIV‐1/2). Exclusion criteria were pregnancy (positive urinary pregnancy screening test), first week of lactation, any kind of immunosuppressive treatment or condition during the last 6 months and severe anaemia (<5 g/dl). Healthy controls were recruited among adults, biologically unrelated to TB patients, living in Kinondoni District. Controls with DM were also recruited in the DM clinic of Mwananyamala Regional Hospital. Eligibility criteria for healthy controls were: HIV negative, no past TB history, absence of symptoms or signs of active TB or other acute infection or major trauma within the last 3 months. Exclusion criteria were identical to active TB cases. Healthy controls were classified as latently infected with *Mtb* based on positive *Mtb*‐specific IFN‐γ enzyme‐linked immunospot assay (ELISPOT) or positive flow cytometry result (frequency of IFN‐γ, TNF‐α, or IL‐2 CD4^+^ T cells after *Mtb*‐specific stimulation >0.03% and >2 times higher than negative control). Healthy control samples without evidence of latent TB infection were excluded from the analysis.

DM disease status was assessed before the start of TB treatment using in parallel (1) the blood glucose testing conducted after an overnight fast of ≥8 h (fasting capillary glucose‐FCG; GlucoPlus™, Diabcare, a plasma‐calibrated glucometer which accuracy conformed to the International Standardization Organization guidelines 25); (2) 2‐h capillary glucose (2‐hCG; standard 75‐g oral glucose tolerance testing); and (3) glycated hemoglobin HbA1c (venipuncture whole blood; immuno‐assay certified by the National Glycohemoglobin Standardization Program and insensitive to hemoglobinopathies (Tina‐quant HbA1c Gen. 2 Cobas Integra 400, Roche Diagnostics, Rotkreuz, Switzerland) 11, 25, 26. DM was defined as repeated measurements of ≥7.0 mmol/l for FCG, ≥11.1 mmol/l for 2‐hCG and ≥6.5% for HbA1c and/or in the presence of history and treatment for DM according to the standard American Diabetes Association and World Health Organization criteria 11, 27, 28. DM status was confirmed after 5 months of TB treatment to exclude volunteers with transient hyperglycaemia related stress induced hyperglycaemia 10. Normal glycaemia was defined as FCG < 6.1 mmol/l and 2‐hCG < 7.8 mmol/l in the absence of treatment for DM. The capillary glucose values were confirmed by plasma testing (Cobas Integra 400 plus). All volunteers with pre‐DM were excluded.

### Study procedures

#### Data and sample collection

Demographic characteristics, health history and symptoms, socioeconomic status (indicators of education, occupation, and wealth using factor analysis) were obtained. Complete blood count was performed (Sysmex XS‐800i automated haematology analyzer). Data were entered directly into an open data kit in a personal digital assistant with real‐time error, range and consistency checks 29. Before TB treatment initiation, PBMC were isolated and frozen to −80°C within 5 h of phlebotomy at a rate of −1°C/min using Nalgene® Mr. Frosty® Cryo 1°C freezing containers before transfer for cryo‐preservation in temperature monitored liquid nitrogen tanks.

#### Immunological assays

Immunological assays were performed after thawing and 5–6 h resting of cryopreserved PBMC. For flow cytometry assays, PBMC (1 × 10^6^) were cultivated overnight in the presence of Brefeldin A (BD GolgiPlug, Becton Dickinson, USA) only (negative control), stimulated with Staphylococcal enterotoxin B (SEB; Sigma, Germany; 200 ng/ml^−1^) (positive control), live *M. bovis* bacilli Calmette‐Guérin vaccine (20 μl BCG Vaccine solution, Staten Serum Institute) or *Mtb* specific peptide pools covering the 10‐kDa culture filtrate antigen (CFP10) and the 6‐kDa early secretory antigen target (ESAT6) protein sequences as described previously (https://www.jpt.com) 30.

#### IFN‐γ ELISPOT assays

ELISPOT was performed as previously described 30. ELISPOT result was defined positive when *Mtb*‐specific stimulation led to ≥55 SFU per 10^6^ cells and fourfold higher than negative control.

#### Intracellular cytokine staining and flow cytometry analyses

Antigen and mitogen stimulated PBMC were incubated in 100 μl PBS containing 1 μl Aqua solution (LIVE/DEAD kit, Invitrogen, USA), and then stained with antibodies specific for CD3 (APC‐H7, SK7, BD Biosciences, USA), CD4 (PE‐CF594, RPA‐T4, BD Biosciences), CD8 (eFluor625NC, RPA‐T8, eBioscience), and CD45RA (BV711, 92430, Biolegend) before fixation, and permeabilization with BD Cytofix/Cytoperm kit (BD Biosciences). PBMCs were then stained with antibodies directed against intracellular expressed IFN‐γ (AF700, B27, BD Biosciences), TNF‐α (PECy7, Mab11, BD Biosciences), IL‐2 (PerCpCy5.5, MQ1‐17H12, Biolegend), IL‐10 (BV 421, JES3‐9D7, Biolegend), IL‐17 (FITC, BL168, Biolegend), IL‐4 (PE, 3010.211, BD Bioscienecs), IL‐5 (PE, TRFK5, Biolegend), IL‐13 (PE, JES10‐5A2, Biolegend), and IL‐9 (Alexa647, MH9A4, BD Biosciences). Cells were fixed and analysed on BD LSRII apparatus equipped with 405, 488, 532, and 633 nm lasers. Gating strategy is shown on Figure S1. Only samples with a cytokine response to SEB at least twice compared to the unstimulated control were considered valid. Following limits of quantitation described for antigen‐specific T cell response measured by intra‐cellular detection of IFN‐γ, TNF‐α, or IL‐2 31, a polychromatic flow cytometry response was considered valid when the frequency of cytokine‐secreting cells after live *M. bovis* BCG or *Mtb*‐specific peptide pool stimulation was above 0.03% and double compared to negative control values for IFN‐γ, TNF‐α, or IL‐2. As such, we retrieved LTBI status from participants for which ELISPOT assay result was missing or returned indeterminate (*n* = 12). Concordance between both assays was high (85.4%). Among discordant cases, four were assigned positive from ELIPOT results and three from flow cytometry results. Among the latters, two samples with 43 and 53 spot forming units already displayed borderline results by ELISPOT.

#### Data analysis

Frequencies of CD4^+^ T cells producing cytokines as well as quantitative cytokine responses were adjusted by subtracting the background level (negative control).

Only cytokines with a flow cytometry responder's frequency ≥50% in at least one study group were kept in subsequent analyses (i.e., IL4/5/13, IL‐9, and IL‐10 were excluded).

Log transformed frequencies of CD4^+^ T cells producing cytokines were analyzed across all participants according to fasting glycaemia by linear regression after adjustment for age and sex. HbA1c results were not considered as they appeared to overestimate DM prevalence in this TB population characterized by high prevalence of anemia and hemoglobinopathies 10.

Statistical analyses were performed using Stata software (StataCorp, College Station, TX, version 12), RStudio (version 0.99.491) and GraphPad Prism 6.

## Availability of Data and Material

The datasets generated and analyzed during the current study are available from the corresponding author on reasonable request.

## Author's Contribution

Noémie Boillat‐Blanco, Claudia Daubenberger, Damien Portevin, and Anneth‐Mwasi N. Tumbo contributed to study conception, study design, study performance, study management, laboratory analyses, data analysis, data interpretation, and manuscript writing. Klaus Reither contributed to study conception, study design, study performance, study management, data interpretation, and critical review of the manuscript. Nicole Probst‐Hensch and Sebastien Gagneux contributed to study conception, study design, study performance, data interpretation, and critical review of the manuscript. Christian Schindler contributed to data analysis, data interpretation, and critical review of the manuscript. Patrizia Amelio, Matthieu Perreau, and Giuseppe Pantaleo contributed to laboratory analyses, interpretation of the data, and critical review of the manuscript. Maliwaza Mganga, and Kaushik L. Ramaiya contributed to interpretation of the data, and critical review of the manuscript. Noémie Boillat‐Blanco had full access to all the data in the study and takes responsibility for the integrity of the data, and the accuracy of the data analysis.

## Ethical Statement

All participants consented in writing to interview and health examination. The Ifakara Health Institute—Institutional Review Board (IHI/IRB/N°:10–2012) and the Medical Research Coordinating Committee of the National Institute for Medical Research (NIMR/HQ/R.8a/Vol.IX/1340), Tanzania, gave ethical clearance.

## Conflicts of Interest

The authors declare no commercial or financial conflict of interest.

## Supporting information

Additional supporting information may be found in the online version of this article at the publisher's web‐site.

**Figure S1**. Gating strategy for the flow cytometry analysis of CD4^+^ T cell cytokine responses.
