# Supplementary figures and images for: Hyperglycaemia is inversely correlated with live M. bovis BCG‐specific CD4+ T cell responses in Tanzanian adults with latent or active tuberculosis
Source: Immun Inflamm Dis. 2018 Apr 11;6(2):345–53. doi: 10.1002/iid3.222 (PMC5946156; doi:10.1002/iid3.222)

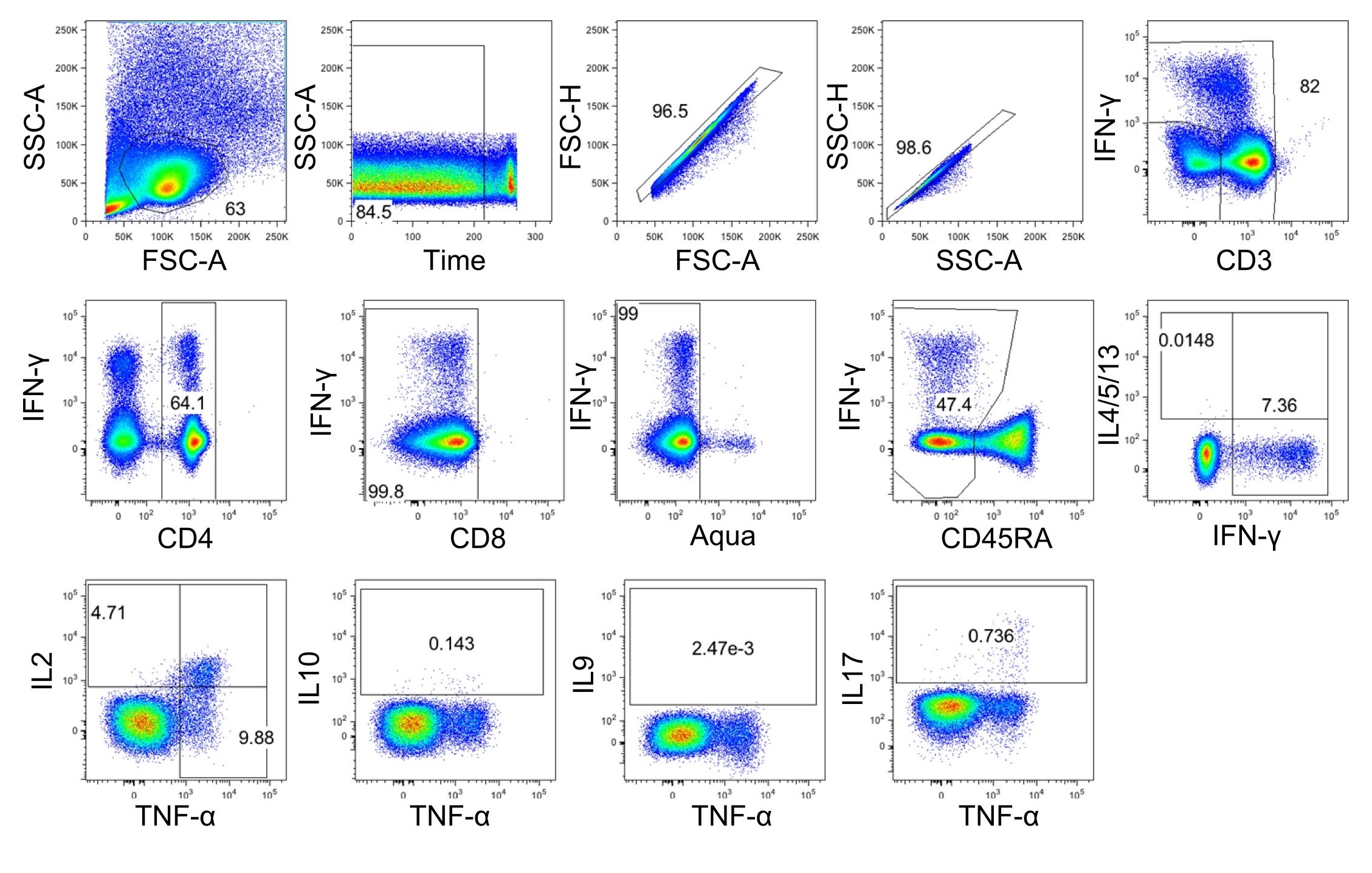

Supplement: Supplementary file 1 — Figure S1. Gating strategy for the flow cytometry analysis of CD4+ T cell cytokine responses. [file IID3-6-345-s001.jpg]
